# Genetic Variants Affecting Iron Metabolism in Healthy Adults: A Systematic Review to Support Personalized Nutrition Strategies

**DOI:** 10.3390/nu16223793

**Published:** 2024-11-05

**Authors:** Elana Sophie Bösch, Jörg Spörri, Johannes Scherr

**Affiliations:** 1 University Centre for Prevention and Sports Medicine, Department of Orthopaedics, Balgrist University Hospital, University of Zurich, 8008 Zurich, Switzerland; 2 Sports Medical Research Group, Department of Orthopaedics, Balgrist University Hospital, University of Zurich, 8008 Zurich, Switzerland

**Keywords:** genetic variants, mineral metabolism, personalized nutrition, SNPs, iron deficiency

## Abstract

**Background/Objectives:** Increased interest in personalized nutrition has led to a growing focus on exploring genetic variants and their impact on nutritional uptake (nutrigenomics). Nevertheless, no systematic review to date has compiled scientific evidence on genetic variants (such as single-nucleotide polymorphisms (SNPs)) affecting mineral metabolism in humans. This review aims to fill this gap and enable optimized personalized nutrition recommendations in health care. **Methods:** Cochrane, Embase and MEDLINE databases were systematically searched for English and German studies published between 2007 and 2023, focusing on genetic variants linked to nutrition. Studies on overweight, diseased, or underage individuals were excluded. Papers with verified findings were assessed for methodological quality using the Joanna Briggs Institute critical appraisal tool. **Results:** Twenty-one scientific papers on SNPs associated with mineral metabolism were included. The majority were observational studies (*n* = 19) conducted on Caucasian populations. Women outnumbered men (37.4%) women, 18.9% men, 43.7% sex not reported. All identified SNPs linked to minerals influenced iron parameters, with the TMPRSS6 gene showing the strongest correlation. Two HFE SNPs (rs1800562 and rs1799945) and one TF SNP (rs1799852) exhibited protective effects, while the other 11 SNPs were linked to increased risk of iron deficiency, suggesting potential benefits from iron supplementation for individuals with those genetic variants. **Conclusions:** This review provides comprehensive insights into the association between genetic variants and mineral metabolism, and the findings highlight the relevance of genetic makeup in optimizing health through nutritional interventions. The generalizability of the findings may be limited to Caucasians, warranting future research with diverse populations. This review was registered with the International Platform of Registered Systematic Review and Meta-Analysis Protocols (INPLASY) on 12 July 2022, under INPLASY202270068 and funded by the University Centre for Prevention and Sports Medicine at Balgrist University Hospital Zurich and the Swiss Innovation Agency Innosuisse, Switzerland.

## 1. Introduction

In an attempt to improve population health and prevent chronic disease onset, governments across the world have introduced general nutritional recommendations (such as the Swiss Food Pyramid [1]). Nutrition is an easily overlooked essential factor in noncommunicable disease mechanisms. Considering the vast diversity of modern populations characterized by varied ancestries and migration backgrounds, the efficacy of previously implemented one-size-fits-all approaches for balanced nutrition has recently been debated [2,3]. The genetic differences between individuals and population groups may affect bioavailability as well as metabolic responses to identical diets [2,3].

Personalized nutrition (PN) uses genetic information, among other patient data, to identify which diet is best suited for an individual or population group [4]. The idea behind PN is that although genes may be set, nutrition is not. Based on genotype, people can gain insight into their metabolism and consequently adapt their nutrition for optimal health, disease prevention or even athletic performance [5]. “Nutrigenomics” is an increasingly used term to describe the combination of nutritional science and genomics. Dorland’s medical dictionary defines nutrigenomics as the field of study “examining how foods affect genes and how individual genetic differences can influence the response to particular nutrients, or other naturally occurring compounds in foods” [6].

Despite being a rather young field, nutrigenomic research has made substantial progress in recent years, revealing many new correlations between genetic variants and metabolism. To the best of our knowledge, no systematic review has investigated associations and knowledge gaps in recent research, underscoring the unmet need for a reliable summary of the current state of knowledge in this field.

The focus of the current systematic review was on genetic variants such as single nucleotide polymorphisms (SNPs) that affect the uptake and bioavailability of minerals, specifically iron. SNPs, occurring approximately every 1000 base pairs, are the most common type of genetic alteration in the human genome and can significantly impact processes such as the metabolism of micro- or macronutrients [2]. In 2019, the global prevalence of anemia among women aged 15–49 years was 31% (95% uncertainty interval: 29, 34), with up to half of cases attributed to dietary iron deficiency [7]. Reducing anemia prevalence is considered vital to improving women’s and children’s health, and a better understanding of the causes will contribute significantly to reducing the prevalence of anemia. By identifying genetic variants that affect iron metabolism (such as bioavailability, requirements, intolerances, or interactions), this review aims to support the use of targeted nutrition and supplementation to improve micronutrient status and consequentially reduce deficiencies. Additionally, this study aims to examine the underlying mechanisms of these variants and to understand whether nutrients might in turn affect the metabolism of individuals with these identified SNPs (nutrigenomics). This information may provide a reliable basis on which health care professionals can make recommendations for personalized nutrition and supplementation.

## 2. Methods

### 2.1. Eligibility Criteria

This review included peer-reviewed studies written in English or German that focused on the relationship between genetic variants and personalized nutrition. The studies included healthy adult subjects of any physical fitness level, ethnicity, or socioeconomic status. Population-based studies that included but were not limited to children or infertile, pregnant, obese, or possibly ill individuals were also considered. To ensure reliable information, this review included only study results that had been successfully validated, meaning their results had been replicated to ensure they were not due to chance. Studies that did not meet these inclusion criteria, were over 15 years old, or did not find significant correlations between genetic alterations and metabolic processes were excluded. Studies using a combined genetic risk score were excluded because they did not allow specific effects to be assigned to individual SNPs. Additionally, papers that examined genetic effects on nutrient intake and preferences were excluded, as these effects could be influenced by various confounding factors rather than solely by genetic causes. Any type of outcome measure or follow-up duration was included in this review (see the full list in Supplementary Materials Table S1).

### 2.2. Data Sources and Search Methods

The search strategy was set up by the first author (EB) and was reviewed and finalized with the help of the coauthors and a medical librarian. The search strategy was designed with the Population, Intervention, Comparison, Outcomes, and Context (PICOC) method and applied to the online databases Embase, MEDLINE and the Cochrane Library (Supplementary Materials Tables S2 and S3) [8]. The Preferred Reporting Items for Systematic reviews and Meta-Analyses (PRISMA) statement was followed for conducting and reporting this review [9]. The search used a combination of key words and MeSH terms (Medical Subject Headings), including but not limited to “genetic variants”, “SNPs”, “personalized nutrition”, “nutrigenomics”, and “genetic association studies”. The final searches were conducted on 13 September 2023 (EB). The study was registered at INPLASY on 12 July 2022 (DOI: 10.37766/inplasy2022.7.0068).

### 2.3. Study Selection and Data Extraction

Studies obtained with the search strategy were screened twice: first for title and abstract and then for the full text. This process was facilitated by using Rayyan, a web-based tool designed to help reviewers screen and select studies [10]. Two reviewers (EB, JSc) independently screened the data. Disagreements were settled through discussion. After deduplication, initial screening, and selection of potential papers from reference lists, 279 papers were screened again to identify studies that were validated. In this process, one reviewer (EB) examined those papers in more detail to finally include only studies whose results were confirmed by at least one other paper. This selection process was peer reviewed.

A customized table was created for data extraction, including study characteristics (authors, date of publication, study type, type of intervention, number of participants, research questions), subject characteristics (age, sex, ancestry, health status), methods of analysis and adjustments and outcome measures (e.g., mean differences, effect sizes, odds ratios). Subsequently, data were extracted by one reviewer (EB).

### 2.4. Risk-of-Bias Assessment

To ensure consistency and rigor in assessing the risk of bias across the included studies, we used the Joanna Briggs Institute (JBI) critical appraisal tool. The checklists consist of 8–10 items assessing various aspects, such as inclusion criteria, cohort and setting descriptions, exposure and outcome measurements and validity, confounding factors, and statistical analysis. The risk of bias assessment was conducted by one author (EB) using the appropriate JBI checklist for each study type: case-control studies (n = 7), cross-sectional studies (n = 11), case series (n = 1), quasi-experimental study (n = 1), and qualitative research study (n = 1). The assessment process was reviewed by one of the authors (JSc) to ensure the validity and reliability of the results. For detailed information on the JBI checklists, please refer to Supplementary Materials Tables S4–S8.

## 3. Results

### 3.1. Study Selection and Characteristics

**Figure 1:**
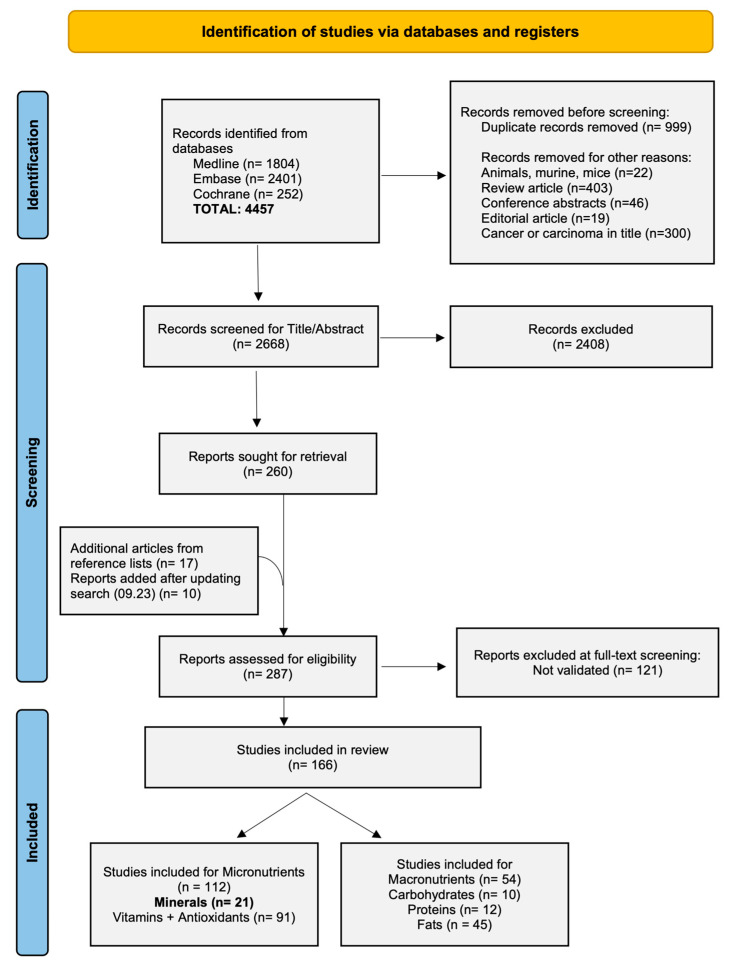
Preferred Reporting Items for Systematic reviews and Meta-analyses (PRISMA) 2020 flow diagram for the study selection.

A total of 4457 papers were retrieved, of which 2668 were screened for eligibility in Rayyan after removing duplicates, animal studies, reviews, conference abstracts, editorials, and studies including ‘cancer’ or ‘carcinoma’ in their title. After title and abstract screening, 275 papers underwent further validation. Of these, 153 papers had results confirmed by at least one other paper and were eligible for review. Among the included studies, 21 focused on associations between genetic variants and mineral metabolism. The PRISMA flow chart (Figure 1) shows the flow of the study selection process.

The 21 included studies involved a total of 22,938 subjects, with a greater proportion of women (n = 8574) than men (n = 4338) and 10,026 subjects of undisclosed gender. The majority of participants were Caucasian (86.1%), followed by Asians (10.1%). Other ethnicities, such as black Africans, Iranians or Arabs, and unspecified ethnicities, accounted for between 1 and 2% in each case. All the papers were published as journal articles and were mainly observational studies, with cross-sectional and case-control designs being the most commonly used approaches for examining genotype distributions and genetic associations.

### 3.2. Study Quality

Methodological quality assessment was conducted by one of the authors using the appropriate JBI checklist [11]. The process was peer reviewed, and discrepancies were resolved through discussion. Summary scores were determined for each study. Unclear or ‘not applicable’ answers received zero points (Supplementary Materials Tables S4–S8). The majority of studies scored 50% or higher, with only one study scoring below this threshold.

### 3.3. SNPs Associated with Minerals (Specifically Iron)

Fourteen SNPs were significantly associated with minerals, specifically with iron parameters, in this review. A complete list of these SNPs is presented in Table 1. The objective of this section is to identify genetic factors influencing iron metabolism and to explore their potential implications for personalized nutrition. Our findings underline the significance of genetic variations in shaping mineral homeostasis and the potential of genetic variations for personalized nutrition and health strategies.

### 3.4. Effects of rs855791 in TMPRSS6

The TMPRSS6 gene variant rs855791 was reported to be significantly associated with markers of iron status, such as ferritin, transferrin, hepcidin and total iron binding capacity (TIBC) [12,13,14,15,16]. Carriers of the risk allele have greater odds of iron deficiency and iron deficiency anemia (IDA), with odds ratios ranging from 1.78 to 22.5.The SNP is more frequent in IDA patients than in healthy control participants [12,15,17,18,19]. The variant was also found to be linked to reduced hemoglobin, mean corpuscular hemoglobin, and mean corpuscular volume, along with increased transferrin levels, all of which are indicators of IDA [13,15,17]. A case study suggested that carrying just one copy of the minor allele A is sufficient to affect IDA risk, with potential interactions involving other TMPRSS6 SNPs [18].

The TMPRSS6 variant was further associated with lower serum iron and ferritin levels in certain populations, increasing the susceptibility of individuals to IDA [12,14,15]. The effect of the variant on iron status is influenced by sex and ancestry. While the association was significant in an Arab female cohort, in Caucasian subjects, the association was significant in men and the population as a whole but not when only women were analysed [14]. Finally, heterozygosity for rs855791 was associated with a 5.0–7.5% increase in red blood cell (RBC) count in IDA patients, indicating blood-related conditions such as iron or vitamin deficiencies [20]. Interestingly, the same study found no association between the variant and hepcidin levels or IDA risk, making it the only study in this review to suggest a protective effect of rs855791 [20]. The findings in the Turkish cohort differ from those observed in subjects of African descent [16,20]. Specifically, this TMPRSS6 variant was negatively associated with TIBC (−11%) and hepcidin levels (−48%) but did not seem to affect dietary iron absorption in the African cohort [16]. Moreover, the absence of homozygotes in the cohort led the authors to question the importance of the SNP on iron status in black African populations [16]. Finally, one study pointed to a high agreement of this variant with rs4820268 and rs2235321 (88.9% and 64.8% agreement, respectively) [17]. In addition to the importance of the variant in diverse ethnic populations, future studies should explore potential interactions between TMPRSS6 SNPs.

### 3.5. Effects of rs4820268 in TMPRSS6

This review revealed that TMPRSS6 SNP rs4820268 (D521D) was associated with various iron parameters in six studies. It has been found to be more prevalent in individuals with IDA than in iron-sufficient control participants, with homozygosity for rs4820268 observed in 29% of IDA patients but not in healthy subjects (*p* < 0.001) [17,18]. Furthermore, carrying the variant allele increased the odds of IDA by between 1.7 and 3.4 times and the odds of iron deficiency by 1.5 times those of noncarriers (Table S9) [15,17]. Similar associations were reported in Chinese cohorts, where the variant allele G was linked to decreased serum iron and TS [15].

Among Caucasian women, heterozygous carriers had nearly 3.5 times greater odds of having low ferritin concentrations (≤8 ng/mL) than did wild-type women [21]. The variant was also positively associated with TIBC, and iron-deficient anemic individuals carrying the rs4820268 risk allele had significantly greater TIBC than did wild-type individuals (heterozygotes: +9.5%, homozygotes: +14.7%) [20]. The opposite observation was made in subjects of black African descent, where homozygotes presented lower TIBC and unsaturated iron-binding capacity (UIBC) values than did wild-type individuals (−14% and −19%, respectively) [16]. Moreover, the GG genotype was associated with 62% lower hepcidin levels, indicating low iron status [16]. Our review revealed an agreement rate of 88.9% between TMPRSS6 SNPs rs4820268 and rs855791. This finding emphasizes the importance of studying SNP combinations in order to gain comprehensive insight into potential joint effects [17].

### 3.6. Effects of rs2235321 in TMPRSS6

Significant associations were identified between the TMPRSS6 SNP rs2235321 (Y739Y) and iron parameters, specifically IDA, in Caucasian cohorts. Women with the SNP had 53.8% lower transferrin saturation (TS) than did those without it [22]. Another study revealed a greater frequency of the variant allele in IDA patients than in iron-normal control participants (52.2% and 24.5%, respectively) [17]. Compared with wild-type individuals, heterozygotes had 90% higher odds to be iron deficient. These findings were supported by a case study on twins with IDA who were heterozygous at this locus [18].

A recent study by Jallow et al. (2021) [16] reported that carriers of minor allele A had 30% lower hepcidin levels than did carriers of wild-type alleles, even after oral iron supplementation (*p* = 0.002). As hepcidin is known to be downregulated in response to low iron levels, decreased hepcidin levels indicate iron deficiency [23]. That study also revealed a positive association between the rs2235321 variant allele and increased values of MCV and MCH, both of which are markers for IDA [16]. Finally, subjects carrying minor allele A had a 17.4% lower baseline UIBC and 13.9% lower total iron-binding capacity (TIBC) (*p* = 0.006 and *p* = 0.000, respectively). Based on these results, rs2235321 may affect hepcidin levels but is unlikely to have a significant impact on dietary iron absorption [16].

### 3.7. Effects of rs2235324 in TMPRSS6

The associations between rs2235324 (also known as K253E) and minerals were examined in two cohorts of patients of Caucasian descent in this review. While 10% of IDA patients were homozygous carriers, none of the healthy control participants exhibited homozygosity, and only 6% carried one copy of the risk allele (*p* = 0.005) [17]. In line with this, the odds of iron deficiency in heterozygotes were nearly nine times greater than those in wild-type individuals [17]. In addition, the SNP was significantly more frequent in iron-deficient women with TS values of 10% or more (*p* = 0.0001) [22]. In summary, heterozygosity for rs2235324 is associated with iron deficiency, suggesting a potential role in iron metabolism among Caucasian populations [16,21].

### 3.8. Effects of rs2413450 in TMPRSS6

The final SNP in TMPRSS6 examined in this review, rs2413450, was associated with iron deficiency markers in two studies [20,24]. Among IDA patients, there was a 26% increase in TIBC in heterozygous carriers compared to wild-type individuals, indicating low iron levels [20,25]. The risk allele was not specified by Batar et al. (2018) [20], but in Caucasian populations, the major allele for this SNP is typically C, while the minor allele is T (MAF = 0.46, allele frequency may vary by ancestry) [26]. A previous study on five iron-refractory iron deficiency anemia (IRIDA) patients identified a mutation near marker D22S1177 on 22q12.3-13.1, which resulted in hepcidin overproduction and impaired iron absorption [24]. Although the reference SNP cluster ID for this mutation could not be identified, its proximity and similar effects suggest that it may be the same SNP studied by Batar et al. (2018) [20]. These findings indicate that this mutation impairs iron binding and transport to cells. However, whether the mutation on 22q12.3-13.1 is identical to rs2413450 has yet to be confirmed. Therefore, further comprehensive research is necessary before this variant can be used for personalized nutrition counselling.

### 3.9. Effects of rs1800562 in HFE

The HFE variant rs1800562 (also known as C282Y) exhibited a significant protective effect against iron deficiency in seven studies included in this review. Consistently, these studies reported reduced serum transferrin levels, supporting the protective role of the minor allele A (coefficient (95% CI): −39.35 (64.67, −14.02), see Table S9) [13,27]. In one study, heterozygotes displayed significantly greater TS (+22.5%, *p* < 0.05) than did wild-type individuals [28]. In another study, rs1800562 heterozygotes were nearly twice as likely to have normal iron levels (66.7% vs. 34.1%) and experienced 83.1% reduced odds of being anemic [13]. Furthermore, heterozygosity for rs1800562 was associated with up to 70% higher ferritin levels in multiple studies, with even stronger effects observed in homozygotes (+293.3% in men and +88.2% in women) [13,14,29]. Notably, the A allele was considerably less frequent in people of Iranian descent than in Caucasians and other ethnic groups (<1% compared to 5.3%) [30,31]. Additionally, in a large genome-wide association study (GWAS), the minor allele A was linked to decreased TIBC and UIBC, as well as increased serum iron levels, further supporting the important and multifaceted impact of rs1800562 on iron parameters [32]. In summary, the significant effect of HFE variant rs1800562 against iron deficiency underscores its impact on iron parameters in Caucasian populations.

### 3.10. Effects of rs1799945 in HFE

This review revealed positive associations of the HFE variant rs1799945 (also known as H63D) with ferritin and TS levels. Compared with carriers of wild-type strains, carriers of this variant had 25.5% to 133% greater ferritin levels and 30% to 136% greater TS [14,28,31]. In a cohort study, male but not female carriers had significantly greater ferritin levels (+9% for CG and +25.5% for GG, *p* = 0.0001), and the association remained significant for the entire cohort when both sexes were considered (β (95% CI): 0.0569 (0.026, 0.085), *p* = 0.003) [14].

In a case control study of hereditary hemochromatosis (HH) patients, all patients were homozygous carriers of the variant and had 2.3 times greater ferritin values than control participants (healthy & wild-type individuals). This increase was observed only in men. The same cohort showed a strong increase in TS (+136%), with no observed sex correlation. However, both of these observations are common symptoms of HH and are therefore not necessarily caused by the SNP [31,33]. Finally, rs1799945 has been negatively associated with transferrin levels, with an 85.6% overall chance of association and a negative mean effect of 15.73 per copy of the minor allele [13,27]. In conclusion, the results suggest a protective effect of rs1799945, meaning that individuals with this SNP are less likely to develop iron deficiencies.

### 3.11. Effects of rs3811647 in TF

The SNP rs3811647 in the TF gene was investigated in six studies, all of which indicated a greater risk for iron deficiency [13,15,27,32,34,35]. Transferrin levels showed a positive correlation in a total of 11 different cohorts, with heterozygotes exhibiting a 7.5% increase and homozygotes exhibiting up to 17.4% higher levels than wild-type individuals [13,27]. Decision tree analyses and chi-square tests confirmed these findings, with wild-type subjects having the lowest transferrin levels, followed by GA heterozygotes and AA homozygotes [13]. In one study, the SNP explained 8.05% of the total variation in transferrin levels, and each A allele had an additive effect of 20.32 [27].

Compared with the wild type, carrying the SNP was associated with a 16.5% lower TS, and the minor allele A was positively associated with TIBC in five cohorts of different ancestry [15,27,32]. However, the SNP was not directly associated with iron deficiency status or anemia risk in the reviewed studies [15,32]. Serum iron was significantly associated with the variant in two populations, but it did not reach significance in the meta-analysis [15]. The variant appeared to affect transferrin properties and TIBC locally without influencing whole-body iron metabolism [32].

Although a negative correlation was observed between rs3811647, serum iron, and serum ferritin (X^2^ = 6.7, *p* = 0.035 and X^2^ = 6.4, *p* = 0.04, respectively), the SNP frequency did not significantly differ between IDA patients and iron-sufficient control participants. This suggests that, while the variant may affect iron status, it is not specifically associated with IDA [35]. In summary, this variant has been consistently positively associated with transferrin levels in different ethnicities as well as been linked to elevated TIBC and reduced TS, ferritin and serum iron concentrations. A better understanding of the underlying mechanisms of this variant is essential to determine its impact and role in targeted nutrition and supplementation.

### 3.12. Effects of rs1799852 in TF

The SNP rs1799852 (also known as L247L) lies on exon 17 of the TF gene and was examined in two instances in this review. The variant showed a strong negative association (78% chance) with serum transferrin levels, with the minor allele having a negative effect of 20.25 (variance explained = 5.5%) [27]. This SNP has been negatively associated with transferrin levels, with a coefficient of −25.45 and 95% confidence interval (−39.29 to −11.61, *p* = 0.0004), and is reported to explain 5.5% of the variation in mean transferrin levels [13,27]. SNPs that reduce serum transferrin typically increase hemoglobin and hematocrit values; however, this variant reduced both hemoglobin and hematocrit values, suggesting potential interference with regular transferrin activity or expression [13].

Furthermore, interaction analysis revealed that homozygous carriers of rs3811647, which are also heterozygous carriers of rs1799852, had 8.3% lower serum transferrin levels than individuals with only rs3811647 (*p* = 0.007) [27]. Finally, in another cohort, rs1799852 was detected in all subjects carrying rs1799899, suggesting a possible linkage between these SNPs [36].

### 3.13. Effects of rs235756 in BMP2

Investigating variant rs235756 in the BMP2 gene, which has previously been associated with hepcidin-induced anemia and iron overload, is essential for an encompassing understanding of iron metabolism disorders [37]. Three papers in this review tested this SNP for associations with iron markers, of which two found significant results.

The SNP was found to be significantly associated with ferritin levels in men (*p* = 0.038); however, the impact of heterozygosity or homozygosity could not be determined due to limited statistical power and sample size [21]. Significant differences in the genotype distribution were observed in an Arab cohort, with nearly 14% of IDA patients being homozygous carriers compared to only 2% of healthy control participants (*p* = 0.05, X^2^ = 5.65) [37]. Additionally, IDA patients presented with abnormally low ferritin levels (<15 ng/mL), suggesting an association between homozygosity at this locus and decreased ferritin levels (*p* = 0.05). Moreover, homozygous carriers had significantly greater odds of being iron deficient anemic, with an odds ratio of 29.3 (95% CI: 1.494, 575.401) and a risk ratio of 7.65 (95% CI: 0.549, 106.47) [37]. In conclusion, the findings regarding this BMP2 gene variant not only underline its relevance to iron metabolism disorders but also provide valuable insights for future research in this field.

### 3.14. Effects of rs2698530 in Chromosome 2

The impact of rs2698530 (in Chr. 2p14) on iron parameters was examined in one of the studies in this review, comprising a GWAS, a replication study and a meta-analysis [32]. The presence of the minor allele C at this locus was positively associated with UIBC in the GWAS, the replication cohort and the meta-analysis, explaining 3% of the total variance with coefficients ranging from 14.25 to 28.75 (Table S9) [32]. The variant also reached nearly genome-wide significance for TIBC in the meta-analysis (*p* = 0.055) and for Log_e_(TS) in the GWAS sample (*p* = 0.12) [32]. In summary, the findings of this review suggest that carrying rs2698530 increases the risk for low iron levels.

## 4. Discussion

This systematic review aimed to identify genetic variants affecting mineral uptake and metabolism, with a specific focus on iron, to support the implementation of precision nutrition and supplementation strategies. In a comprehensive analysis of 21 studies, this review identified associations between 14 SNPs and iron parameters or disorders of iron metabolism. Among these SNPs, five were found in TMPRSS6 alone, exhibiting consistent associations with iron markers. The TMPRSS6 gene is crucial for iron homeostasis, and subjects carrying variant alleles in this gene demonstrated increased odds of IDA, with rs855791 increasing the odds of IDA up to 22.5 times [12]. The variable effects of the TMPRSS6 variant rs4820268 on iron parameters depending on carrier ancestry emphasize the importance of considering allele frequencies to better assess the importance of a SNP in different populations. For example, the HFE variant rs1800562 is very uncommon in Asian and Middle Eastern people when compared with Caucasians, and the SNP is therefore more relevant for applied use in Caucasian populations [30]. Rs1800562 and rs1799945 in TMPRSS6 and rs1799852 in TF were the only SNPs that were found to exert a protective effect against iron deficiency in this review; however, further research, especially on rs1799945 and rs1799852, is needed to confirm their associations with iron metabolism as well as their interactions with one another and evaluate the impact of these variants on nutritional interventions.

Contrasting observations were made for the TF variant rs3811647, which was consistently associated with increased transferrin levels across diverse populations. However, despite numerous studies demonstrating associations, it cannot be concluded from this review that rs3811647 has a direct regulatory effect on iron metabolism, and further research on the mode of action is warranted, not least because the variant’s effect was also found to interact with SNPs in other genes [27]. Similarly, rs235756 was associated with serum ferritin levels but not with other iron markers, such as hemoglobin, serum iron, RBC or platelet count, or with iron deficiency or anemia risk, preventing definitive conclusions on the importance of the variant for personalized nutrition [15,37].

This systematic review builds on existing knowledge by providing a thorough overview of SNPs significantly linked to mineral metabolism, particularly iron metabolism, in healthy adults. In this way, our findings offer valuable insights for developing personalized nutrition strategies. Including diverse populations provides valuable insights into how genetic mutations affect iron metabolism across various ancestries while highlighting the need for further research on broader demographic groups. This review not only emphasizes the importance of different SNPs related to iron metabolism when implementing personalized nutrition but also sets the groundwork for personalized approaches, addressing mineral deficiencies and optimizing nutritional interventions. Finally, the genetic interactions observed in this review underscore the importance of studying frequent combinations of SNPs rather than singular SNP effects. As highlighted by Zeisel (2020), the current research often examines the impact of individual gene variants when metabolic traits typically arise from polygenic interactions. Understanding patterns of variants would therefore be valuable for effective nutritional treatments and prevention [38].

A few limitations should be considered. While no exclusions were made based on the ethnicity of the study populations, the overrepresentation of Caucasian cohorts (86%) in this review restricts the generalizability of findings to minority populations. Biases may have been introduced due to the broad research scope and exclusion of subjects with a BMI > 25 kg/m^2^. BMI may influence responses to supplementation regardless of genotype, which makes the inclusion of overweight individuals important in the context of personalized nutrition [39]. Furthermore, we have not included any study results on epigenetic investigations in the analyses. This is because it is known from epigenetic studies that environmental factors influence cell properties and the activity status of genes and thus regulate possible mechanisms of action at the cellular level (DOI: 10.1016/j.jtemb.2023.127203). However, as there is very little literature available, we have not evaluated this topic further. Additionally, the lack of studies on minerals other than iron suggests a research gap or possibly a flaw in our search strategy. More recent studies have identified associations among genetic variations and other minerals, such as copper (rs35691438, rs671), magnesium, potassium, and sodium. These findings highlight the importance of further research in these areas to gain a more comprehensive understanding of the interplay between genetics and mineral metabolism [40,41]. Moreover, our review was restricted to studies published within the last 15 years (2007–2023), in line with the criteria established in our review protocol (DOI: 10.37766/inplasWy2022.7.0068).This focus on more recent research might have excluded earlier studies, which could have provided additional insights into genetic variants linked to mineral metabolism. While earlier work on this topic has been extensively reviewed elsewhere, the decision to limit the time frame was made for consistency and clarity in discussing contemporary findings. Finally, inconsistent genetic notations complicate comparisons of results across studies, highlighting the need for a standardized notation system in genetic studies.

To achieve an optimal personalized approach to supplementation, it is essential to measure iron metabolism at both serum protein level (e.g., serum ferritin, transferrin saturation) and through epigenetic analysis, in addition to genetic testing. This comprehensive assessment will enable effective monitoring of supplementation effects and allow for a truly personalized approach.

In conclusion, all but three variants in this review (rs1800562, rs1799945 and rs1799852) are associated with a greater risk of iron deficiency, suggesting that iron supplementation may benefit individuals carrying these variants. Although the exact underlying mechanisms are not fully understood for many SNPs, this review emphasizes the importance of both individual effects of different SNPs and interactions between genetic variants in shaping personalized nutrition interventions. The presented findings have practical implications for medical practitioners in the design of personalized nutrition programs tailored to individual genetic profiles. With the knowledge compiled in this review, practitioners can consider integrating genetic testing into their practice to help identify individuals at risk of iron deficiency and develop more personalized dietary recommendations. Nutrition plans that incorporate genetic profiles, combined with personal health data, environmental influences, and lifestyle factors, may offer a promising approach for improving iron status in populations at risk for deficiencies [5]. This study serves as a comprehensive foundation for future research and contributes to the growing knowledge of how genetic information can inform targeted nutrition therapy.

## Figures and Tables

**Table 1:** SNPs with verified effects on minerals and their associated genes.

SNP	Gene	Affected Iron Parameter	Odds Ratios for ID/IDA
rs855791 (V736A)	*TMPRSS6*	Ferritin, Transferrin, TS, Hemoglobin, MCV/MCH, Hepcidin, TIBC, Serum iron, RBC	1.78–22.5
rs4820268 (D521D)	*TMPRSS6*	Ferritin, TS, Hepcidin, UIBC, TIBC, Serum iron	1.5/1.7–3.4
rs2235321 (Y739Y)	*TMPRSS6*	TS, MCV/MCH, Hepcidin, UIBC, TIBC	1.9
rs2235324 (K253E)	*TMPRSS6*	TS	8.7
rs2413450	*TMPRSS6*	TIBC	Not reported
rs1800562 (C282Y)	*HFE*	Ferritin, Transferrin, TS, UIBC, TIBC, Serum iron	0.169
rs1799945 (H63D)	*HFE*	Ferritin, Transferrin, TS,	Not reported
rs3811647	*TF*	Ferritin, Transferrin, TS, TIBC, Serum iron	Not reported
rs1799852 (L247L)	*TF*	Transferrin, Haemoglobin	Not reported
rs235756	*BMP2*	Ferritin	29.3
rs2698530	*N/A (Chromosome 2)*	UIBC TIBC	Not reported

ID: iron deficiency; IDA: iron deficiency anemia; TS: transferrin saturation; MCV: mean corpuscular volume; MCH: mean corpuscular hemoglobin; UIBC: unsaturated iron-binding capacity; TIBC: total iron-binding capacity.

## Data Availability

No new data were created or analyzed in this study.

## Supplementary Materials

The following supporting information can be downloaded at: https://www.mdpi.com/article/10.3390/nu16223793/s1, Table S1: Inclusion and exclusion criteria. Table S2: PICOC methodology. Table S3: Search strategies for the systematic literature search. Table S4: Case-Control Studies. Table S5: Cross-Sectional Studies. Table S6: Case Series. Table S7: Quasi-Experimental Studies. Table S8: Qualitative Research. Table S9: Iron parameter study.
