# Supplementary material for: Genetic Variants Affecting Iron Metabolism in Healthy Adults: A Systematic Review to Support Personalized Nutrition Strategies
Source: Nutrients. 2024 Nov 5;16(22):3793. doi: 10.3390/nu16223793 (PMC11597267; doi:10.3390/nu16223793)
Supplement: Supplementary file 1 [file nutrients-16-03793-s001.zip › nutrients-3234733-s1-8.pdf]

Supplementary – intended for publication

Search Strategy

Table S1: Inclusion and exclusion criteria

Inclusion Criteria	*Exclusion Criteria*
• Peer-reviewed primary studies<br>• Languages: English or German<br>• Subjects: healthy adults of any physical fitness, ethnicity, or socioeconomic status<br>• Focused on the aspect of personalized nutrition and genetic variations (e.g. SNPs)<br>• any type of nutrition-based observation and intervention<br>• any outcome measure or follow-up duration	• Animal studies<br>• Children <18 years<br>• Diseased, pregnant, or infertile subjects<br>• Overweight & obese subjects (BMI >25 kg/m^2)<br>• Papers published prior to 2007<br>• Non-validated studies<br>• Studies with nonsignificant correlations<br>• Studies using genetic risk scores<br>• Studies on alcohol intake, food intake and food preferences.<br>• Reviews, meta-analyses, conference abstracts

Table S2: PICOC methodology

PICOC	*Description*
***P**opulation*	adult participants of any age and physical fitness
***I**ntervention*	any types of nutrition-based observation and interventions
***C**omparison*	controlled quantitative studies (any type of controls)
***O**utcome*	any kind of outcome measures and lengths of follow-up
***C**ontext*	clinical and nutrition context

Table S3: Search strategies for the systematic literature search

Database	*Date*	*Concepts*	*Results*
Cochrane	First search: 30.05.2022	(('single nucleotide' OR gen* OR allel* OR homozygo* OR heterozygo*) NEAR/3 (polymorphism* OR varia* OR pattern* OR diversit* OR mutation* OR differentiat* OR divergenc*)):ti,ab,kw OR ((nutritional* NEAR/3 (genomic* OR genetic*)):ti,ab,kw) OR nutrigenomic*:ti,ab,kw OR<br><br>AND<br><br>((personal* OR precis* OR individuali* OR tailored OR custom*) NEAR/3 (nutri* OR macronutri* OR micronutri* OR alimentary OR metabolism* OR diet* OR feed* OR food* OR intake* OR uptake*)):ti,ab,kw OR ((nutri* OR macronutri* OR micronutri* OR alimentary OR diet* OR feed* OR food*) NEAR/20 (intake* OR uptake* OR advice* OR intervention* OR choice* OR recommend*)):ti,kw OR (daily NEAR/20 (intake* OR	252

	Last search: 05.09.2023	uptake*)):ti,kw OR ((deficien* OR defective* OR insufficien*) NEAR/20 (nutri* OR macronutri*<br><br>AND<br><br>((gen* OR allel* OR homozygo* OR heterozygo*) NEAR/4 (interact* OR affect* OR modulat* OR influenc* OR associat* OR correlate* OR determinant* or based OR linkage* OR diathes* OR predisposition* OR prognos* OR propensit* OR proneness* OR anticipation* OR<br><br>Filter:<br>Cochrane Library publication date Between Jan 2007 and Dec 2022	276

EMBASE	First search: 30.05.2022	'single nucleotide polymorphism'/exp OR 'genetic variability'/exp OR 'genetic variation'/de OR 'nutrigenomics'/exp OR 'nutrigenetics'/exp OR ((('single nucleotide' OR gen* OR allel* OR homozygo* OR heterozygo*) NEAR/3 (polymorphism* OR varia* OR pattern* OR diversit* OR mutation* OR differentiat* OR divergenc*)):ti,ab,kw) OR ((nutritional* NEAR/3 (genomic* OR genetic*)):ti,ab,kw) OR nutrigenomic*:ti,ab,kw OR nutrigenetic*:ti,ab,kw<br><br>AND<br><br>'personalized nutrition'/exp OR 'dietary intake'/exp/mj OR 'food intake'/exp/mj OR 'nutritional deficiency'/exp/mj OR 'nutrient uptake'/exp OR (((personal* OR precis* OR individuali* OR tailored OR custom*) NEAR/3 (nutri* OR macronutri* OR micronutri* OR alimentary OR metabolism* OR diet* OR feed* OR food* OR intake* OR uptake*)):ti,ab,kw) OR (((nutri* OR macronutri* OR micronutri* OR alimentary OR diet* OR feed* OR food*) NEAR/20 (intake* OR uptake* OR advice* OR intervention* OR choice* OR recommend*)):ti,kw) OR ((daily NEAR/20 (intake* OR uptake*)):ti,kw) OR (((deficien* OR defective* OR insufficien*) NEAR/20 (nutri* OR macronutri* OR micronutri* OR alimentary OR diet* OR feed* OR food*)):ti,kw)<br><br>AND<br><br>‘gene interaction'/exp OR 'genetic association'/exp OR 'genetic association study'/exp OR 'genetic predisposition'/de OR 'genetic susceptibility'/exp OR (((gen* OR allel* OR homozygo* OR heterozygo*) NEAR/4 (interact* OR affect* OR modulat* OR influenc* OR associat* OR correlate* OR determinant* OR based OR linkage* OR diathes* OR predisposition* OR prognos* OR propensit* OR proneness* OR anticipation* OR susceptibilit*)):ti,ab,kw)<br><br>AND<br><br>([english]/lim OR [german]/lim) AND [2007-2022]/py NOT	2401

	Last search: 05.09.2023	[conference abstract]/lim NOT ([animals]/lim NOT [humans]/lim) NOT ('juvenile'/exp NOT 'adult'/exp)	2564
MEDLINE (OVID)	First search: 30.05.2022<br><br>Last search: 13.09.2023	exp Polymorphism, Single Nucleotide/ or Genetic Variation/ or exp Genomic Structural Variation/ or exp Nutrigenomics/ or (('single nucleotide' or gen* or allel* or homozygo* or heterozygo*) adj3 (polymorphism* or varia* or pattern* or diversit* or mutation* or differentiat* or divergenc*)).ti,ab,kw. or (nutritional* adj3 (genomic* or genetic*)).ti,ab,kw. or nutrigenomic*.ti,ab,kw. or nutrigenetic*.ti,ab,kw.<br><br>AND<br><br>exp *Diet/ or exp *Dietary Supplements/ or exp *Eating/ or exp *Deficiency Diseases/ or ((personal* or precis* or individuali* or tailored or custom*) adj3 (nutri* or macronutri* or micronutri* or alimentary or metabolism* or diet* or feed* or food* or intake* or uptake*)).ti,ab,kw. or ((nutri* or macronutri* or micronutri* or alimentary or diet* or feed* or food*) adj20 (intake* or uptake* or advice* or intervention* or choice* or recommend*)).ti,kw. or (daily adj20 (intake* or uptake*)).ti,kw. or ((deficien* or defective* or insufficien*) adj20 (nutri* or macronutri* or micronutri* or alimentary or diet* or feed* or food*)).ti,kw.<br><br>AND<br><br>exp Genetic Association Studies/ or Genetic Predisposition to Disease/ or ((gen* or allel* or homozygo* or heterozygo*) adj4 (interact* or affect* or modulat* or influenc* or associat* or correlate* or determinant* or based or linkage* or diathes* or predisposition* or prognos* or propensit* or proneness* or anticipation* or susceptibilit*)).ti,ab,kw. limit to ((english or german) and yr="2007 - 2022")<br><br>AND<br><br>NOT (animals not humans).sh. NOT ((exp infant/ OR exp child/ OR adolescent/) NOT exp adult/)	1804<br><br>1975

Quality Assessment: JBI Critical Appraisal Tools

Y (yes)
N (No)
U (unclear)
N/A (not applicable)

Table S4: Case-Control Studies

Source / Question	Were the groups comparable other than the presence of disease in cases or the absence of disease in controls?	Were cases and controls matched appropriately?	Were the same criteria used for identification of cases and controls?	Was exposure measured in a standard, valid and reliable way?	Was exposure measured in the same way for cases and controls?	Were confounding factors identified?	Were strategies to deal with confounding factors stated?	Were outcomes assessed in a standard, valid and reliable way for cases and controls?	Was the exposure period of interest long enough to be meaningful?	Was appropriate statistical analysis used?	**Overall Rating**
Meidtner et al., 2018	y	y	y	u	y	y	y	y	N/A	y	8/10
McLaren et al., 2011	y	y	y	y	y	y	y	y	N/A	y	9/10
Poggiali et al., 2015	n	u	u	y	y	y	y	u	N/A	y	5/10
Batar, et al., 2018	y	y	y	y	y	n	y	y	N/A	y	8/10
Al-Amer et al., 2020	y	y	y	y	y	n	n	y	N/A	y	7/10
Pei et al., 2014	u	u	y	y	y	n	n	y	N/A	y	5/10
Al-Amer et al., 2021	y	u	u	y	y	n	n	y	N/A	y	5/10

Table S5: Cross-Sectional Studies

Source / Question	Were the criteria for inclusion in the sample clearly defined?	Were the study subjects and the setting described in detail?	Was the exposure measured in a valid and reliable way?	Were objective, standard criteria used for measurement of the condition?	Were confounding factors identified?	Were strategies to deal with confounding factors stated?	Were the outcomes measured in a valid and reliable way?	Was appropriate statistical analysis used?	**Overall Rating**
Blanco-Rojo et al., 2011	y	y	y	y	y	u	y	y	7/8
Beiranvand et al., 2015	n	y	y	y	n	n	y	y	5/8
Baeza-Richer et al., 2015	n	y	u	y	n	u	n	y	3/8
Aranda et al., 2010	y	y	u	N/A	y	y	y	y	6/8
Sarriá et al., 2007	n	y	y	y	y	y	y	y	7/8
Kutalik et al., 2011	y	u	y	u	y	y	y	u	5/8
Al-Amer et al., 2018	y	y	y	y	n	n	y	u	4/8
Lee et al., 2012	y	y	y	y	n	u	y	y	6/8
Ji et al., 2018	n	n	y	y	n	y	y	y	5/8
Terada et al., 2009	y	y	y	y	y	y	y	y	8/8
An et al., 2012	y	y	y	y	y	y	y	y	8/8

Table S6: Case Series

Source / Question	Were there clear criteria for inclusion in the case series?	Was the condition measured in a standard, reliable way for all participants included in the case series?	Were valid methods used for identification of the condition for all participants included in the case series?	Did the case series have consecutive inclusion of participants?	Did the case series have complete inclusion of participants?	Was there clear reporting of the demographics of the participants in the study?	Was there clear reporting of clinical information of the participants?	Were the outcomes or follow up results of cases clearly reported?	Was there clear reporting of the presenting site(s)/clinic(s) demographic information?	Was statistical analysis appropriate?	**Overall Rating**
Pinto et al., 2017	y	u	u	N/A	y	y	y	y	y	N/A	6/10

Table S7: Quasi-Experimental Studies

Source / Question	Is it clear in the study what is the 'cause' and what is the 'effect' (i.e. there is no confusion about which variable comes first)?	Were the participants included in any comparisons similar?	Were the participants included in any comparisons receiving similar treatment/care, other than the exposure or intervention of interest?	Was there a control group?	Were there multiple measurements of the outcome both pre and post the intervention/exposure?	Was follow up complete and if not, were differences between groups in terms of their follow up adequately described and analyzed?	Were the outcomes of participants included in any comparisons measured in the same way?	Were outcomes measured in a reliable way?	Was appropriate statistical analysis used?	**Overall Rating**
Jallow et al., 2021	y	y	y	y	n	u	y	y	y	7/9

Table S8: Qualitative Research

Source / Question	Is there congruity between the stated philosophical perspective and the research methodology?	Is there congruity between the research methodology and the research question or objectives?	Is there congruity between the research methodology and the methods used to collect data?	Is there congruity between the research methodology and the representation and analysis of data?	Is there congruity between the research methodology and the interpretation of results?	Is there a statement locating the researcher culturally or theoretically?	Is the influence of the researcher on the research, and vice-versa, addressed?	Are participants, and their voices, adequately represented?	Is the research ethical according to current criteria or, for recent studies, and is there evidence of ethical approval by an appropriate body?	Do the conclusions drawn in the research report flow from the analysis, or interpretation, of the data?	**Overall Rating**
Melis et al., 2008	u	y	y	y	y	N/A	N/A	u	n	y	5/10
