# Supplementary material for: Genetic Variants Affecting Iron Metabolism in Healthy Adults: A Systematic Review to Support Personalized Nutrition Strategies
Source: Nutrients. 2024 Nov 5;16(22):3793. doi: 10.3390/nu16223793 (PMC11597267; doi:10.3390/nu16223793)
Supplement: Supplementary file 1 [file nutrients-16-03793-s001.zip › nutrients-3234733-Table S9.pdf]

Gene and SNP	*Source*	*Iron parameter*														*Outcome*					***Risk of bias***	***Notes***
		Ferritin	*Transferrin*	*TS %*	*Hematocrit*	*Hemoglobin*	*MCV, MCH*	*Hepcidin*	*UIBC*	*TIBC*	*Serum Iron*	*Genotype frequency*	*Iron deficiency/ IDA risk*	*RBC count*	*Ancestry*	*Absolute Values and Mean Differenc e*	*Fact or*	*Effect size*	*Other*	*P-value*	*JBI assessm ent*	
**TMPRSS6** rs855791	Batar et al., 2018													**x**	Caucasian (Turks)	*Mean ± SD [$10^6 mm^3$]*<br><br>Wt: 4.0 ± 0.5<br>Het.: 4.3 ± 0.6<br>Hom.: 4.2 ± 0.6	Wt: 1<br>Het.: 1.075<br>Hom.: 1.050	Het.: *Cohen's d* = 0.055		Het.: P = 0.03<br>Hom.: NS	8/10	These values refer to IDA subjects, associations in iron normal controls were not significant
	Baeza-Richer et al., 2015		**x**												Caucasian			*Linear regression for SNP (TT)*<br>Coef (CI 95%): 15.43 (0.07, 30.78)		0.049	3/8	
	Meidtner et al., 2018 [†]	**x**													Caucasian	*Mean (95%CI), [pmol/L]*<br><br><u>Men</u><br>GG (Wt): 320.9 (307.0, 335.5)<br>AG: 302.2 (291.4, 313.4)<br>AA: 285.9 (269.8, 303.0)	GG: 1<br>AG: 0.942<br>AA: 0.891	<u>Overall</u><br>β (95% CI): 0.031 (0.009, 0.052)<br><u>Men</u><br>β (95% CI): 0.057 (0.023, 0.091)		Overall: 0.0058<br>Men: 0.0010	8/10	Effect estimates are given for a change in standardized log-transformed ferritin per A allele<br><br>Associations for women were not significant

Gene and SNP	Source	Iron parameter														Outcome					Risk of bias	Notes
		Ferritin	*Transferrin*	*TS %*	*Hematocrit*	*Hemoglobin*	*MCV, MCH*	*Hepcidin*	*UIBC*	*TIBC*	*Serum Iron*	*Genotype frequency*	*Iron deficiency/ IDA risk*	*RBC count*	*Ancestry*	*Absolute Values and Mean Difference*	*Factor*	*Effect size*	*Other*	*P-value*	*JBI assessment*	
**TMPRSS6** rs855791	Al-Amer et al., 2020	x													Arabs				*Genotype distribution, [n]*<br>Normal value<br>CC (Wt): 1<br>CT: 48<br>TT: 1<br>Low value<br>CC: 0<br>CT: 45<br>TT: 13<br>X^2= 10.85	0.004	7/10	Low value: ferritin < 15 ng/ml
											x								*Genotype distribution, [n]*<br>Normal value<br>CC (Wt): 1<br>CT: 36<br>TT: 1<br>Low value<br>CC: 0<br>CT: 57<br>TT: 13<br>X^2= 7.18	0.028		Iron deficiency: ferritin < 15 ng/ml & haemoglobin > 12 g/dl
													x						*Odds and risk for IDA for TT compared to CC + CT*<br>OR (95%CI): 22.5 (2.496, 204.836)<br>RR (95%CI): 5.53 (0.897, 34.114)	0.005		

Gene and SNP	*Source*	***Iron parameter***														***Outcome***					***Risk of bias***	***Notes***
		Ferritin	*Transferrin*	*TS %*	*Hematocrit*	*Hemoglobin*	*MCV, MCH*	*Hepcidin*	*UIBC*	*TIBC*	*Serum Iron*	*Genotype frequency*	*Iron deficiency/ IDA risk*	*RBC count*	*Ancestry*	*Absolute Values and Mean Difference*	*Factor*	*Effect size*	*Other*	*P-value*	*JBI assessment*	
**TMPRSS6** rs855791	An et al., 2012												x		Asian (Chinese)				*Odds for Iron deficiency, OR (95%CI)* 1.55 (1.40, 1.70)<br><br>*Odds for IDA, OR (95%CI)* 1.78 (1.59, 1.79)	4.96E-8 *<br><br>8.43E-9 *	8/8	Iron deficiency: TS < 16% or serum ferritin < 15 ng/ml or FEP/hemoglobin ratio > 3.0 mg/g<br><br>IDA: hemoglobin < 120 g/L<br><br>* Bonferroni corrected<br><br>[a] Associations for hemoglobin were not significant in the Zhuang cohort
											x							*Association of the A allele, β (SE)* HanE: 1.41 (0.24) HanN: - 1.45 (0.4) Zhuang: - 1.23 (0.36) Meta-analysis: - 1.37 (0.18)		6.90E-9<br>3.70E-4<br>7.76E-4<br>8.28 E-14 *		
						x												*Association of the A allele,* β (SE) [a] HanE: - 2.31 (0.59) HanN: - 2.65 (1.20) Meta-analysis: -1.99 (0.46)		1.01E-4<br>0.03<br>6.51 E-5 *		

Gene and SNP	*Source*	***Iron parameter***														***Outcome***					***Risk of bias***	***Notes***
		Ferritin	*Transferrin*	*TS %*	*Hematocrit*	*Hemoglobin*	*MCV, MCH*	*Hepcidin*	*UIBC*	*TIBC*	*Serum Iron*	*Genotype frequency*	*Iron deficiency/ IDA risk*	*RBC count*	*Ancestry*	*Absolute Values and Mean Difference*	*Factor*	*Effect size*	*Other*	*P-value*	*JBI assessment*	
**TMPRSS6** rs855791				**x**														*Association of the A allele, β (SE)* HanE: - 0.02 (0.004) HanN: - 0.03 (0.01) Zhuang: - 0.02 (0.007) Meta-analysis: - 0.02 (0.003)		9.92E-10 9.74E-5 8.39E-4 4.47E-15 *		
	Pinto et al., 2017												**x**		Caucasian				Observed IRIDA twins to be heterozygous carriers		6/10	
	Jallow et al., 2021									**x**					Black-African	*Mean ± SE [μmol/L]* GG (Wt): 68.29 ± 1.81 GA: 60.79 ± 3.5	Wt: 1 GA: 0.890	β = - 7.5		0.036	7/9	GA has 89% lower average TIBC than Wt per risk allele (A), TIBC reduced by 7.5 units.
								**x**								*Mean ± SE [ng/mL]* GG (Wt): 9.5 ± 2.3 GA: 4.96 ± 1	Wt: 1 GA: 0.522			0.015		Aboslute values are estimated based on Fig. 4A in Jallow et al. (2021).No values were given for the AA genotype

Gene and SNP	Source	*Iron parameter*														*Outcome*					*Risk of bias*	*Notes*
		Ferritin	*Transferrin*	*TS %*	*Hematocrit*	*Hemoglobin*	*MCV, MCH*	*Hepcidin*	*UIBC*	*TIBC*	*Serum Iron*	*Genotype frequency*	*Iron deficiency/ IDA risk*	*RBC count*	*Ancestry*	*Absolute Values and Mean Difference*	*Factor*	*Effect size*	*Other*	*P-value*	*JBI assessment*	
**TMPRSS6** rs855791	Poggiali et al., 2015					x									Caucasian	*Mean difference [g/dL]*<br><br>Wt: Ref.<br>Het..: -0.5 g/dL<br>Hom.: -0.8 g/dL				0.08<br>0.01	5/10	no absolute values were reported by Poggiali et al. (2015)
							x									*Mean difference*<br><br>MCV *[fL]*<br>Wt: Ref.<br>Het.: -5.0<br>Hom.: -5.1<br>MCH *[pg]*<br>Wt: Ref.<br>Het.: -1.7<br>Hom.:-2.01				0.001<br>0.002<br><br>0.006<br>0.003		

Gene and SNP	*Source*	***Iron parameter***													*Ancestry*	***Outcome***					***Risk of bias***	***Notes***
		Ferritin	*Transferrin*	*TS %*	*Hematocrit*	*Hemoglobin*	*MCV, MCH*	*Hepcidin*	*UIBC*	*TIBC*	*Serum Iron*	*Genotype frequency*	*Iron deficiency/ IDA risk*	*RBC count*		*Absolute Values and Mean Difference*	*Factor*	*Effect size*	*Other*	*P-value*	*JBI assessment*	
**TMPRSS6** rs855791	Poggiali et al., 2015											**x**			Caucasian				*IDA status by genotype*<br>IDA<br>Wt: 22%<br>Het.: 42.5%<br>Hom.: 25.5%<br>Control<br>Wt: 55%<br>Het: 45%<br>Hom.: 0%	*For Homozygotes*<br>X^2 P-value < 0.001	5/10	Odds for heterozygous SNP carriers to have IDA compared to wild types.<br><br>P-value expresses significant difference in homozygous genotype frequency between IDA patients and iron-normal controls
													**x**						*Odds for heterozygotes to have IDA*<br>OR (95%CI): 3.70 (1.18, 11.46)	N/R		
	Pei et al., 2014											**x**			Asian				*IDA status by genotype*<br>IDA<br>TT (Wt): 25.4%<br>TC:62.7 %<br>CC: 11.9%<br>Control<br>TT (Wt): 28%<br>TC: 46.7%<br>CC: 25.2%	Freq. distribution of all genotypes<br>P = 0.06<br><br>Freq. Distribution of CC<br>P = 0.03	5/10	"The frequency distribution of the TT, TC, and CC genotypes in both groups showed borderline difference. The proportion of C homozygotes was significantly lower in IDA patients."

Gene and SNP	Source	Iron parameter													Ancestry	Outcome					Risk of bias	Notes
		Ferritin	Transferrin	TS %	Hematocrit	Hemoglobin	MCV, MCH	Hepcidin	UIBC	TIBC	Serum Iron	Genotype frequency	Iron deficiency/ IDA risk	RBC count		Absolute Values and Mean Difference	Factor	Effect size	Other	P-value	JBI assessment	
**TMPRSS6** rs855791	Pei et al., 2014												x						*Odds for CC to have IDA (compared to TT + TC)* OR (95%CI): 0.4 (0.17, 0.95)	0.04	5/10	

Footnotes

Effect directions refer to minor allele A (T on reverse DNA strand); reference genotype: GG (wt) (CC on reverse strand)

Allele frequencies: G=0.564810, A=0.435190, T=0.000000

Calculations to obtain Cohen's d are presented in the *Calculation*s sheet

† Statistical analyses in this study were performed on log-transformed values.

Abbreviations: Wt, wild type; Het., heterozygotes; hom., homozygotes; MD, mean difference; NS, not significant; SE, standard error; SD, standard deviation; $^{x}2$ Chi-square test; IRIDA iron refractory iron deficiency anemia; MCV, mean corpuscular volume; MCH, mean corpuscular haemoglobin

Gene and SNP	Source	Ferritin	Transferrin	TS %	Hematocrit	Hemoglobin	MCV, MCH	Hepcidin	UIBC	TIBC	Serum Iron	Genotype frequency	Iron deficiency/ IDA risk	RBC count	Ancestry	Absolute Values and Mean Difference	Factor	Effect size	Other	P-value	JBI assessment	Notes
**TMPRSS6** rs4820268	An et al., 2012										x				Asian (Chinese)			*Association of the G allele, β (SE)* HanE: -1.20 (0.59) HanN: -1.41 (0.4) Zhuang: -1.38 (0.37) Meta-analysis: - 1.28 (0.18)		8.31E-7 5.45E-4 2.23-4 6.42E-12 *	8/8	Iron deficiency: TS < 16% or serum ferritin < 15 ng/ml or FEP/hemoglobin ratio > 3.0 mg/g<br><br>IDA: hemoglobin < 120 g/L<br><br>* Bonferroni corrected
				x														*Association of the G allele, β (SE)* HanE: -0.02 (0.004) HanN: -0.02 (0.01) Zhuang: -0.03 (0.01)		1.63E-6 0.001 7.66E-5 5.31E-10 *		

Gene and SNP	Source	Iron parameter														Outcome					Risk of bias	Notes
		Ferritin	*Transferrin*	*TS %*	*Hematocrit*	*Hemoglobin*	*MCV, MCH*	*Hepcidin*	*UIBC*	*TIBC*	*Serum Iron*	*Genotype frequency*	*Iron deficiency/ IDA risk*	*RBC count*	*Ancestry*	*Absolute Values and Mean Difference*	*Factor*	*Effect size*	*Other*	*P-value*	*JBI assessment*	
																		Meta-analysis: - 0.02 (0.003)				
**TMPRSS6** rs4820268	An et al., 2012												**x**						*Odds for Iron deficiency, OR (95%CI)* 1.53 (1.38, 1.68)<br><br>*Odds for IDA, OR (95%CI)* 1.72 (1.54, 1.91)	9.00E-8 *<br><br>6.49E-8 *	8/8	
	Poggiali et al., 2015											**x**			Caucasian				*IDA status by genotype* <u>IDA</u> Wt: 25% Het.: 46% Hom.: 29% <u>Control</u> Wt: 55% Het: 45% Hom.: 0%	*For Homozygotes* X^2 P-value < 0.001	5/10	P-value expresses significant difference in homozygous genotype frequency between IDA patients and iron-normal controls
													**x**						*Odds for heterozygotes to have IDA* OR (95%CI): 3.40 (1.10, 4.71)	N/R		

Gene and SNP	Source	Iron parameter													Ancestry	Outcome					Risk of bias	Notes
		Ferritin	*Transferrin*	*TS %*	*Hematocrit*	*Hemoglobin*	*MCV, MCH*	*Hepcidin*	*UIBC*	*TIBC*	*Serum Iron*	*Genotype frequency*	*Iron deficiency/ IDA risk*	*RBC count*	*Ancestry*	*Absolute Values and Mean Difference*	*Factor*	*Effect size*	*Other*	*P-value*	*JBI assessment*	
**TMPRSS6** rs4820268	Pinto et al., 2017												x		Caucasian				Observed IRIDA twins to be heterozygous carriers		6/10	
	Batar et al., 2018									x					Caucasian (Turks)	*Mean ± SE [µg/dL]*<br><br>Wt: 342.6 ± 120.8<br>Het.: 375.3 ± 68.6<br>Hom.: 392.9 ± 59.3<br><br>*Mean difference, MD ± SD*<br>Wt.: Ref.<br>Het.: 32.7 ± 822.96<br>Hom.: 50.3 ± 421.22	Wt: 1<br>Het.: 1.095<br>Hom.: 1.147	Het.: *Cohen's d* = 0.0397<br>Hom.: *Cohen's d* = 0.119		0.04<br>0.03	8/10	
	Jallow et al., 2021							x							Black-African	*Mean ± SE [ng/mL]*<br><br>AA (Wt): 9.5 ± 2.5<br>GA: 3.6 ± 0.8<br>GG: 3.3 ± 0.7	Wt: 1<br>GA: 0.379<br>GG: 0.374			GG vs. Wt<br>P = 0.002	7/9	Absolute values are estimated based on Fig. 4A in Jallow et al., 2021

Gene and SNP	*Source*	***Iron parameter***													*Ancestry*	***Outcome***					***Risk of bias***	***Notes***
		Ferritin	*Transferrin*	*TS %*	*Hematocrit*	*Hemoglobin*	*MCV, MCH*	*Hepcidin*	*UIBC*	*TIBC*	*Serum Iron*	*Genotype frequency*	*Iron deficiency/ IDA risk*	*RBC count*		*Absolute Values and Mean Difference*	*Factor*	*Effect size*	*Other*	*P-value*	*JBI assessment*	
**TMPRSS6** rs4820268	Jallow et al., 2021								**x**						Black-African	*Mean ± SE [µmol/L]*<br><br>AA (Wt): 53.76 ± 2.27<br>AG: 50.47 ± 3.64<br>GG: 43.51 ± 3.61	Wt: 1<br>GA: 0.939<br>GG: 0.809	AA: Ref.<br>AG: β = - 3.29<br>GG: β = - 10.25		P = 0.368 (NS)<br>P = 0.005	7/9	
										**x**						*Mean ± SE [µmol/L]*<br><br>AA (Wt): 68.29 ± 1.81<br>AG: 63.33 ± 2.98<br>GG: 58.76 ± 2.86	Wt: 1<br>GA: 0.927<br>GG: 0.860	AA: Ref.<br>AG: β= - 4.96<br>GG: β= - 9.54		P = 0.089 (NS)<br>P = 0.001		

Gene and SNP	*Source*	***Iron parameter***													*Ancestry*	***Outcome***					***Risk of bias***	***Notes***
		Ferritin	*Transferrin*	*TS %*	*Hematocrit*	*Hemoglobin*	*MCV, MCH*	*Hepcidin*	*UIBC*	*TIBC*	*Serum Iron*	*Genotype frequency*	*Iron deficiency/ IDA risk*	*RBC count*		*Absolute Values and Mean Differenc e*	*Fact or*	*Effect size*	*Other*	*P-value*	*JBI assessm ent*	
**TMPRSS6** rs4820268	Ji et al., 2018	x													Caucasian			*Odds for low ferritin, OR(95%C I)* [a]<br>AG: 3.443 (1.312, 9.021)	*Genotypes by Ferritin status Values for women only*<br><br>AA (Wt)<br>Low ferritin: n= 6<br>Medium ferritin: n= 93<br>High ferritin: n= 10<br>AG<br>Low ferritin: n= 27<br>Medium ferritin: n= 125<br>High ferritin: n= 20<br>GG<br>Low ferritin: n= 7<br>Medium ferritin: n= 60<br>High ferritin: n= 10	Genotyp e freq.: P = 0.025 [b]<br><br>OR: P = 0.012	5/8	Ferritin<br>low ≤ 8ng/mL<br>medium 8-60ng/mL<br>high >60ng/mL<br><br>[a] Value after adjusting for significant donor parameters; Pre-adjustment OR (95%CI): 3.348 (1.328-8.438), P = 0.010<br><br>[b] P-value after adjusting for significant donor parameters; Pre-adjustment P = 0.043
Footnotes<br>Effect directions refer to minor allele G; reference genotype: AA (wt)<br>Allele frequencies: G=0.459037, A=0.540963, C=0.000000<br>Cohen's d represents the nr. of SD that hepcidin levels in heterozygous carrieres deviate drom the mean levels in Wt. (after controling for the variability (SE)).<br>Calculations to obtain Cohen's d are presented in the *Calculation*s sheet																						
**TMPRSS6** rs2235321	Lee et al., 2012			x											Caucasian	*Mean ± SD [%]*<br><br>Wt: 13 ± 16<br>SNP: 6 ± 4<br><br>MD: - 7 ± 10	Wt: 1<br>SNP: 0.46 2	*Cohen's d* = - 0.424		0.0021	6/8	
												x							SNP more frequent in subjects with TS < 10%	0.0135		

Gene and SNP	Source	Iron parameter														Outcome					Risk of bias	Notes
		Ferritin	Transferrin	TS %	Hematocrit	Hemoglobin	MCV, MCH	Hepcidin	UIBC	TIBC	Serum Iron	Genotype frequency	Iron deficiency/ IDA risk	RBC count	Ancestry	Absolute Values and Mean Difference	Factor	Effect size	Other	P-value	JBI assessment	
**TMPRSS6** rs2235321	Poggiali et al., 2015											x			Caucasian				*IDA status by genotype*<br>IDA<br>Wt: 47.8%<br>Het.: 40.7%<br>Hom.: 11.5%<br>Control<br>Wt: 75.5%<br>Het: 24.5%<br>Hom.: 0%	*For Homozygotes*<br>X^2 P-value = 0.004	5/10	P-value expresses significant difference in homozygous genotype frequency between IDA patients and iron-normal controls
													x						*Odds for Heterozygotes to be IDA*<br>OR (95%CI): 1.90 (0.72, 5.09)			
	Pinto et al., 2017												x		Caucasian				Observed IRIDA twins to be heterozygous carriers		6/10	
	Jallow et al., 2021						x									*Mean ± SE*<br><br>MCV *[fL]*<br>GG (Wt): 77.8 ± 1.54<br>AA: 83.7 ± 1.79<br>MCH *[pg]*<br>GG (Wt): 26.4 ± 0.60<br>AA: 29.0 ± 0.70	MCV<br>GG: 1<br>AA: 1.076<br>MCH<br>GG: 1<br>AA: 1.098	GG: Ref.<br><br>MCV<br>AA: β = 5.81<br>MCH<br>AA: β = 2.55		P = 0.002<br><br>P = 0.000	7/9	Differences between Wt and heterozygotes (GA) were not significant.

Gene and SNP	Source	Iron parameter													Ancestry	Outcome					Risk of bias	Notes
		Ferritin	*Transferrin*	*TS %*	*Hematocrit*	*Hemoglobin*	*MCV, MCH*	*Hepcidin*	*UIBC*	*TIBC*	*Serum Iron*	*Genotype frequency*	*Iron deficiency/ IDA risk*	*RBC count*		*Absolute Values and Mean Difference*	*Factor*	*Effect size*	*Other*	*P-value*	*JBI assessment*	
**TMPRSS6** rs2235321								**x**							Black-African	*Mean ± SE [ng/mL]*<br><br>GG (Wt): 9.5 ± 2.4<br>GA: 4.2 ± 0.8<br>AA: 6.6 ± 1.5	Wt: 1<br>GA: 0.442<br>AA: 0.695			P = 0.035		Absolute values are estimated based on Figure 4A in Jallow et al., 2021
									**x**							*Mean ± SE [µmol/L]*<br><br>GG (Wt): 53.76 ± 2.27<br>GA: 45.59 ± 3.83<br>AA: 44.40 ± 3.30	Wt: 1<br>GA: 0.848<br>AA: 0.826	GG: Ref.<br>GA: β = -8.17<br>AA: β = -9.36		P = 0.036<br>P = 0.006	7/9	
										**x**						*Mean ± SE [µmol/L]*<br><br>GG (Wt): 68.29 ± 1.81<br>GA: 61.63 ± 3.06<br>AA: 58.78 ± 2.63	Wt: 1<br>GA: 0.902<br>AA: 0.861	GG: Ref.<br>GA: β = -6.66<br>AA: β = -9.51		P = 0.032<br>P = 0.000		

<u>Footnotes</u>

Effect directions refer to minor allele A; reference genotype GG (wt)

Allele frequencies: G=0.636033, A=0.363967, C=0.000000

Calculations to obtain Cohen's d are presented in the *Calculations* sheet

Abbreviations: Wt, wild type; het., heterozygotes; MD, mean difference; MCV, mean corpuscular volume; MCH, mean corpuscular haemoglobin

Gene and SNP	Source	Iron parameter														Outcome					Risk of bias	Notes
		Ferritin	Transferrin	TS %	Hematocrit	Hemoglobin	MCV, MCH	Hepcidin	UIBC	TIBC	Serum Iron	Genotype frequency	Iron deficiency/ IDA risk	RBC count	Ancestry	Absolute Values and Mean Difference	Factor	Effect size	Other	P-value	JBI assessment	
**TMPRSS6** rs2235324	Lee et al., 2012			x											Caucasian				SNP more frequent in subjects with TS ≥ 10%	0.0001	6/8	
	Poggiali et al., 2015											x			Caucasian				*IDA status by genotype*<br>IDA<br>Wt: 52%<br>Het.: 38%<br>Hom.: 10%<br>Control<br>Wt: 94%<br>Het: 6%<br>Hom.: 0%	*For Homozygotes*<br>X^2 P-value = 0.005	5/10	P-value expresses significant difference in homozygous genotype frequency between IDA patients and iron-normal controls
													x						*Odds for heterozygotes to have IDA*<br>OR (95%CI): 8.70 (2.01, 37.91)	N/R		
Footnotes<br>Effect directions refer to minor allele C; reference genotype TT (Wt).<br>Allele frequencies: T=0.619381, C=0.380619, G=0.000000<br>Abbreviations: Wt, wild type; het., heterozygotes; OR, odds ratio																						
**TMPRSS6** rs2413450	Batar et al., 2018									x					Caucasian	*Mean ± SE for IDA subjects, [µg/dL]*<br>Wt: 330.6 ± 132.2<br>Het.: 416.6 ± 53.1<br>MD ± SD: 86 ± 771.26	Wt: 1<br>Het.: 1.260	*Cohen's d* = 0.112		0.04	8/10	

Gene and SNP	*Source*	***Iron parameter***													*Ancestry*	***Outcome***					***Risk of bias***	***Notes***
		Ferritin	*Transferrin*	*TS %*	*Hematocrit*	*Hemoglobin*	*MCV, MCH*	*Hepcidin*	*UIBC*	*TIBC*	*Serum Iron*	*Genotype frequency*	*Iron deficiency/ IDA risk*	*RBC count*		*Absolute Values and Mean Difference*	*Factor*	*Effect size*	*Other*	*P-value*	*JBI assessment*	
	Melis et al., 2008											**x**			Caucasian				SNP observed in IRIDA patients		5/10	

<u>Footnotes</u>
Effect directions refer to minor allele T; reference genotype CC (Wt)
Allele frequencies: C=0.546368, T=0.453632
Calculations to obtain Cohen's d are presented in the *Calculation*s sheet
Abbreviations: Wt, wild type; het., heterozygotes; MD, mean difference

Gene and SNP	*Source*	*Ferritin*	*Transferrin*	*TS %*	*Hematocrit*	*Hemoglobin*	*MCV, MCH*	*Hepcidin*	*UIBC*	*TIBC*	*Serum Iron*	*Genotype frequency*	*Iron deficiency/ IDA risk*	*RBC count*	*Ancestry*	*Absolute Values and Mean Difference*	*Factor*	*Effect size*	*Other*	*P-value*	*JBI assessment*	*Notes*
**HFE** rs1800562	Terada et al., 2009	**x**													274 Caucasians + 234 non-Caucasians	*Median (IQR) [µg/L]*<br>GG (Wt): 94.9 (57.8, 158.2)<br>GA: 162.1 (69.6, 232.2)	Wt: 1<br>GA: 1.708			0.06	8/8	
	Meidtner et al., 2018 †	**x**													Caucasian	*Mean (SD) [pmol/L]*<br><u>Men</u><br>GG (Wt): 301.0 (293.2, 309.1)<br>GA: 336.1 (309.5, 365.0)<br>AA: 1'183.7 (654.8, 2,139.7)<br><u>Women</u><br>GG (Wt): 118.8 (116.3,	<u>Men</u><br>WT: 1<br>GA: 1.117<br>AA: 3.933<br><u>Women</u><br>WT: 1<br>GA: 1.125<br>AA: 1.882	<u>All</u><br>β (95%CI): 0.165(0.116, 0.213)<br><u>Men</u><br>β (95%CI): 0.188 (0.11, 0.266)<br><u>Women</u><br>β (95%CI): 0.149 (0.087, 0.212)		3.81E-11<br>2.08E-6<br>3.08E-6	8/10	EPIC cohort (n= 9347)

Gene and SNP	*Source*	***Iron parameter***													*Ancestry*	***Outcome***					***Risk of bias***	***Notes***
		Ferritin	*Transferrin*	*TS %*	*Hematocrit*	*Hemoglobin*	*MCV, MCH*	*Hepcidin*	*UIBC*	*TIBC*	*Serum Iron*	*Genotype frequency*	*Iron deficiency/ IDA risk*	*RBC count*		*Absolute Values and Mean Difference*	*Factor*	*Effect size*	*Other*	*P-value*	*JBI assessment*	
**HFE** rs1800562																121.4)<br>GA: 133.7 (125.0, 142.9)<br>AA: 223.6 (146.9, 340.3)						
	Baeza-Richer et al., 2015 †	x													Caucasian			*Linear regression for SNP (GA) and logFerritin*<br>Coef (95%CI): 0.17 (0.00, 0.34)		0.048	3/8	Parameter: LogFerritin - transformed due to skewedness
			x															*Linear regression for SNP (GA)*<br>Coef (95%CI): -39.35 (64.67, -14.02)		0.0025		

Gene and SNP	Source	Iron parameter													Ancestry	Outcome					Risk of bias	Notes
		Ferritin	Transferrin	TS %	Hematocrit	Hemoglobin	MCV, MCH	Hepcidin	UIBC	TIBC	Serum Iron	Genotype frequency	Iron deficiency/ IDA risk	RBC count		Absolute Values and Mean Difference	Factor	Effect size	Other	P-value	JBI assessment	
**HFE** rs1800562													x						*Odds for GA to have abnormal iron* [a]<br>OR (95%CI): 0.169 (0.058, 0.490)	0.0011	3/8	[a] Back-step logistic regression<br>OR informs of the probability of disease: being anaemic vs. deficient/normal and anaemic/deficient vs. Normal<br><br>Iron deficiency: Hb ≥ 12 g/dL and Ferritin < 20 µg/L or Hb ≤ 12 g/dL and Ferritin > 20 µg/L<br><br>IDA: Hb < 12 g/dL and Ferritin < 20µg/L
	McLaren et al., 2011								x						Caucasian			GWAS<br>Coef: -44.27<br>Follow-up<br>Coef: -42.7	Meta-analysis<br>Variance explained: 4.1%	GWAS: 3.15E-7<br>Follow-up: 0.0004<br>Meta-analysis: 5.72E-10*	9/10	* genome-wide corrected P = 0.00019
										x					Caucasian			GWAS<br>Coef: -35.36<br>Follow-up<br>Coef: -21.58	Meta-analysis<br>Variance explained: 3.4%	GWAS: 1.31E-7*<br>Follow-up: 0.035<br>Meta-analysis: 2.68E-8**	9/10	* genome-wide corrected P = 0.043<br>** genome-wide corrected P = 0.0089

Gene and SNP	*Source*	***Iron parameter***														***Outcome***					***Risk of bias***	***Notes***
		Ferritin	*Transferrin*	*TS %*	*Hematocrit*	*Hemoglobin*	*MCV, MCH*	*Hepcidin*	*UIBC*	*TIBC*	*Serum Iron*	*Genotype frequency*	*Iron deficiency/ IDA risk*	*RBC count*	*Ancestry*	*Absolute Values and Mean Difference*	*Factor*	*Effect size*	*Other*	*P-value*	*JBI assessment*	
**HFE**<br>rs1800562											x							GWAS<br>Coef: 9.07<br>Follow-up<br>Coef: -21.16	Meta-analysis<br>Variance explained: 1.9%	GWAS: 0.0091<br>Follow-up: 0.0005<br>Meta-analysis: 1.93E-5	9/10	
	Blanco-Rojo et al., 2011		x												Caucasian			Effect per A allele: -44.87	100% probability of association<br><br>Variance explained: 3.07 %		7/8	
	Beiranvand et al., 2015											x			Iranian				In contrast to Caucasian populations, the frequency of rs1800562 was even smaller (0.008 - 0%) in Asian and Middle Eastern people. SNP might have negible effect in these populations.		5/8	
	Aranda et al., 2010			x											Caucasian	*Mean ±SD [%]*<br><br>GG (Wt): 26.7 ± 11.2<br>GA: 32.7 ± 11.9<br>MD ± SD: 6 ± 16.34	Wt: 1<br>GA: 1.225	Multilinear regression<br>All: β (SE) = 5.75 (2.75)<br>Women: β (SE) = 6.43 (3.03)		Mean values<br>P < 0.05<br><br>MLR<br>All: 0.037<br>Women: 0.035	6/8	

Gene and SNP	Source	Iron parameter													Ancestry	Outcome					Risk of bias	Notes
		Ferritin	Transferrin	TS %	Hematocrit	Hemoglobin	MCV, MCH	Hepcidin	UIBC	TIBC	Serum Iron	Genotype frequency	Iron deficiency/ IDA risk	RBC count	Ancestry	Absolute Values and Mean Difference	Factor	Effect size	Other	P-value	JBI assessment	
**HFE**<br>rs1800562 x rs1799945	Aranda et al., 2010			x											Caucasian	*Mean ± SD [%]*<br><br>Men<br>Wt: 37.7 ± 20.8<br>Het.: 53.4 ± 19<br>MD ±SD: 15.7 ± 28.17<br>Women<br>Wt: 26.7 ± 11.2<br>Het.: 40.5 ± 5.4<br>MD ± SD: 13.8 ± 12.43	Men<br>Wt: 1<br>Het.: 1.416<br><br>Women<br>Wt: 1<br>Het.: 1.517	Men<br>*Cohen's d* = 0.56<br>Women<br>*Cohen's d* = 1.11<br><br>Multilinear regression<br>*Whole cohort:*<br>β (SE)= 16.4 (4.2)<br>*Women:*<br>β (SE)= 13.11 (5.59)<br>*Men:*<br>β (SE)= 13.21 (6.59)		Mean values<br>Men P < 0.05<br>Women P< 0.01<br><br>MLR<br>All: P < 0.001<br>Women: P = 0.02<br>Men: P = 0.045	6/8	
		x																Multilinear regression<br>*Whole cohort:*<br>β(SE) = 0.32 (0.18)		0.049		

Footnotes
Effect directions refer to minor allele A; reference genotype: GG (wt)
Allele frequencies: G=0.947281, A=0.052719
† Statistical analyses in this study were performed on log-transformed ferritin values.
Abbreviations: IQR, interquartile range (25th, 75th percentile); Wt, wild type; MD, mean difference; SD, standard deviation

Gene and SNP	Source	Iron parameter													Ancestry	Outcome					Risk of bias	Notes
		Ferritin	Transferrin	TS %	Hematocrit	Hemoglobin	MCV, MCH	Hepcidin	UIBC	TIBC	Serum Iron	Genotype frequency	Iron deficiency/ IDA risk	RBC count		Absolute Values and Mean Difference	Factor	Effect size	Other	P-value	JBI assessment	
<u>Footnotes</u><br>Effect directions refer to heterozygosity for rs1800562 and rs1799945; reference: wild types for both SNPs.<br>Calculations to obtain Cohen's d are presented in the *Calculations* sheet<br>Abbreviations: Wt, wild type; het., heterozygotes; MD, mean difference																						
rs1800562 x rs1375515	Baeza-Richer et al., 2015 †	x													Caucasian	*Mean ± SD [ng/mL]*<br><br>rs1375515: AA (Wt) or AG<br>Rs1800562:<br>GG (Wt): 1.182 ± 0.384<br>GA: 1.377 ± 0.336	AX / GG: 1<br>AX / GA: 1.165			0.027	3/8	Iron parameter: LogFerritin
<u>Footnotes</u><br>Effect directions refer to heterozygosity(GA) for rs1800562 simultaneously as genotypes AA/GA in rs1375515; reference: GG (Wt) for rs1800562<br>Effect directions refer to minor allele A; reference genotype: GG (wt)<br>† Statistical analyses in this study were performed on log-transformed ferritin values.<br>Abbreviations: Wt, wild type; SD, standard deviation																						
**HFE** rs1799945	Meidtner et al., 2018 †	x													Caucasian	*Mean (95%CI), [pmol/L]*<br><br><u>Men</u><br>CC (Wt): 296.7 (288.0, 305.7)<br>CG: 323.3 (307.4, 340.0)<br>GG: 372.4 (317.3, 437.0)	Wt: 1<br>CG: 1.090<br>GG: 1.255	<u>Overall</u><br>β = 0.056 95% CI (0.026, 0.085)<br><u>Men</u><br>β = 0.097 95% CI (0.050, 0.145)		Sex-diff.: < 0.05<br><br>0.003<br><br>0.0001	8/10	EPIC cohort (n= 9347)

Gene and SNP	Source	Iron parameter													Ancestry	Outcome					Risk of bias	Notes
		Ferritin	*Transferrin*	*TS %*	*Hematocrit*	*Hemoglobin*	*MCV, MCH*	*Hepcidin*	*UIBC*	*TIBC*	*Serum Iron*	*Genotype frequency*	*Iron deficiency/ IDA risk*	*RBC count*	*Ancestry*	*Absolute Values and Mean Difference*	*Factor*	*Effect size*	*Other*	*P-value*	*JBI assessment*	
**HFE** rs1799945	Beiranvand et al., 2015	**x**													Iranian	*Mean(SD), [ng/mL]*<br><br>Wt (Healthy): 119.53 ± 46.75<br>SNP (HH patients): 278.6 ± 63	Wt: 1<br>SNP: 2.331		Mean level of serum ferritin in HH men was 3x higher than healthy men *<br>CI: 152.62 ± 196.14	* P ≤ 0.001	5/8	
				**x**												*Mean ± SD [%]* (only men)<br><br>Wt (Healthy): 37.83 ± 9.5<br>SNP (HH patients): 89.56 ± 17.32	Wt: 1<br>SNP: 2.367		TS level was higher in HH patients without being affected by gender and it was also higher than the normal range (up to 45%) *<br>CI: 44.55 ± 64.31	* P ≤ 0.001		
	Aranda et al., 2010			**x**											Caucasian	*Mean ± SD [%] values only of women*<br><br>Wt: 26.7 ± 11.2<br>Het.: 30.6 ± 14.5<br>Hom.: 34.9 ± 13.1	Wt: 1<br>Het.: 1.146<br>Hom.: 1.307	Het. Cohen's *d* = 0.213<br>Hom. Cohen's *d* = 0.476<br><br><u>MLR</u><br>Het.: β (SE) = 3.00 (1.24)<br>Hom.: β (SE) = 7.26 (2.66)		< 0.01<br>< 0.01<br><br>0.015<br>0.006	6/8	

Gene and SNP	*Source*	***Iron parameter***														***Outcome***					***Risk of bias***	***Notes***
		Ferritin	*Transferrin*	*TS %*	*Hematocrit*	*Hemoglobin*	*MCV, MCH*	*Hepcidin*	*UIBC*	*TIBC*	*Serum Iron*	*Genotype frequency*	*Iron deficiency/ IDA risk*	*RBC count*	*Ancestry*	*Absolute Values and Mean Difference*	*Factor*	*Effect size*	*Other*	*P-value*	*JBI assessment*	
**HFE** rs1799945	Blanco-Rojo et al., 2011		**x**												Caucasian			Effect per G allele: -15.73	85.6% probability of association<br><br>Variance explained: 2.40 %		7/8	
	Baeza-Richer et al., 2015		**x**												Caucasian			*Linear regression for CG + GG* Coef (95%CI): -16.79 (-29.64, -3.95)		0.011	3/8	

Footnotes
Effect directions refer to minor allele G; reference genotype: CC (wt)
Allele frequencies: C=0.863913, G=0.136087
† Statistical analyses in this study were performed on log-transformed values.
Calculations to obtain Cohen's d are presented in the *Calculations* sheet
Abbreviations: HH hereditary hemochromatosis, CI, confidence interval

**TF** rs3811647	Blanco-Rojo et al., 2011			**x**											Caucasian	*Mean ± SD [%]*<br><br>GG (Wt): 21.8 ± 10.9<br>AA + AG: 18.2 ± 8.7<br>MD ± SD: - 3.6 ± 13.96	Wt: 1<br>AA + AG: 0.835	*Cohen's d* = - 0.258		0.007	7/8	

Gene and SNP	Source	Iron parameter													Ancestry	Outcome					Risk of bias	Notes
		Ferritin	Transferrin	TS %	Hematocrit	Hemoglobin	MCV, MCH	Hepcidin	UIBC	TIBC	Serum Iron	Genotype frequency	Iron deficiency/ IDA risk	RBC count		Absolute Values and Mean Difference	Factor	Effect size	Other	P-value	JBI assessment	
**TF**<br>rs3811647			x												Caucasian	*Mean ± SD [mg/dL]*<br><br>GG (Wt): 292.2 ± 48.7<br>GA: 314.6 ± 51.6<br>AA: 342.2 ± 67.3	Wt: 1<br>GA: 1.077<br>AA: 1.171	GA Cohen's *d* = 0.316<br>AA Cohen's *d* = 0.602<br><br>Effect per A allele: 20.32	100% probability of association<br><br>Variance explained: 8.08 %	<u>Mean values</u><br>P < 0.001		
	Kutalik et al., 2011 [†]		x												Caucasian			CH: β = 0.362<br>AUSI: β = 0.358<br>AUSII: β = 0.353<br>Meta-analysis: β (SE) = 0.358 (0.029)		1.27E-10<br>5.25E-19<br>8.80E-9<br>1.45E-35	5/8	
	Baeza-Richer et al., 2015		x												Caucasian	*Mean ± SD [mg/dL]*<br><br>GG (Wt): 302.039 ± 54.097<br>GA: 324.634 ± 55.870<br>AA: 354.571 ± 62.296	Wt: 1<br>GA: 1.075<br>AA: 1.174	GA Cohen's *d* = 0.2905<br>AA Cohen's *d* = 0.636	*Linear regression*<br>GA: Coef (95%CI): 21.29 (8.74, 33.84)<br>AA: Coef (95%CI): 51.48 (30.88, 72.02)	< 0.0001	'3/8	

Gene and SNP	*Source*	***Iron parameter***													*Ancestry*	***Outcome***					***Risk of bias***	***Notes***
		Ferritin	*Transferrin*	*TS %*	*Hematocrit*	*Hemoglobin*	*MCV, MCH*	*Hepcidin*	*UIBC*	*TIBC*	*Serum Iron*	*Genotype frequency*	*Iron deficiency/ IDA risk*	*RBC count*		*Absolute Values and Mean Difference*	*Factor*	*Effect size*	*Other*	*P-value*	*JBI assessment*	
**TF** rs3811647	An et al., 2012		**x**												Asian (Chinese			*Association for A allele, β (SE)* HanE: 18.46 (1.68) HanN: 16.42 (3.36) Zhuang: 17.61 (1.91) Meta-analysis: 17.88 (1.18)		8.18 E-27 1.53E-6 4.86E-19 3.77E-51*	8/8	* Bonferroni corrected<br><br>HanE, HanN & Zhuang represent the different cohorts studied. The Meta-analysis contains the pooled sample.<br><br>[a] Associations for HuanN & Meta-analysis were not significant.
											**x**							*Association for A allele, β (SE)* [a] HanE: 0.52 (0.25) - 0.042 Zhuang: -0.76 (0.34)		0.042 0.03		

Gene and SNP	*Source*	***Iron parameter***														***Outcome***					***Risk of bias***	**Notes**
		Ferritin	*Transferrin*	*TS %*	*Hematocrit*	*Hemoglobin*	*MCV, MCH*	*Hepcidin*	*UIBC*	*TIBC*	*Serum Iron*	*Genotype frequency*	*Iron deficiency/ IDA risk*	*RBC count*	*Ancestry*	*Absolute Values and Mean Difference*	*Factor*	*Effect size*	*Other*	*P-value*	*JBI assessment*	
**TF** rs3811647										**x**								*Association for A allele, β (SE)* HanE: 3.71 (0.41) HanN: 3.56 (0.79) Zhuang: 3.70 (0.52) Meta-analysis: 3.68 (0.30)		7.49E-19 8.57E-6 4.21E-12 3.40E-34*		
	McLaren et al., 2011									**x**					Caucasian			GWAS Coef: 21.49 Follow-up Coef: 16.69	Meta-analysis variance explained: 4.2%	GWAS: 0.0023 Follow-up: 0.012 Meta-analysis: 0.00015	9/10	
	Al-Amer et al., 2018										**x**				Arabic				*Genotype distribution, [n]* Iron normal GG (Wt): 24 GA: 14 Low Iron GG (Wt): 26 GA: 44 X^2: 6.7	0.035	4/8	IDA: Ferritin < 15ng/mL and Hb < 12g/dL<br><br>Iron deficiency: Ferritin < 15ng/mL and Hb > 12g/dL

Gene and SNP	Source	Iron parameter														Outcome					Risk of bias	Notes
		Ferritin	Transferrin	TS %	Hematocrit	Hemoglobin	MCV, MCH	Hepcidin	UIBC	TIBC	Serum Iron	Genotype frequency	Iron deficiency/ IDA risk	RBC count	Ancestry	Absolute Values and Mean Difference	Factor	Effect size	Other	P-value	JBI assessment	
**TF** rs3811647		x																	*Genotype distribution, [n]*<br>Iron normal<br>GG (Wt): 34<br>GA: 18<br>Low Iron<br>GG (Wt): 23<br>GA: 33<br>X^2: 6.4	0.04		

Footnotes

Effect directions refer to minor allele A; reference genotype: GG (wt)

Allele frequencies: G=0.666694, A=0.333306

Calculations to obtain Cohen's d are presented in the *Calculations* sheet

† The phenotypes were log-transformed prior to association analyses, normalized to have zero mean & unit variance and adjusted for the effects of sex, age, self-reported smoking status, alcohol intake and principal components

Abbreviations: MD, mean difference; X^2 Chi-square test

Gene and SNP	Source	Ferritin	Transferrin	TS %	Hematocrit	Hemoglobin	MCV, MCH	Hepcidin	UIBC	TIBC	Serum Iron	Genotype frequency	Iron deficiency/ IDA risk	RBC count	Ancestry	Absolute Values and Mean Difference	Factor	Effect size	Other	P-value	JBI assessment	Notes
**TF** rs3811647 x rs1799852	Blanco-Rojo et al., 2011		x												Caucasian	*Mean ± SD [mg/dL]*<br>GG (Wt) + CC (Wt): 301.4 ± 46.5<br>GG + CT: 276.4 ± 48.7<br>MD ± SD: - 25 ± 67.3	GG + CC: 1<br>GG + CT: 0.917	*Cohen's d* = - 0.37		0.007	7/8	

Footnotes

Calculations to obtain Cohen's d are presented in the *Calculations* sheet

Abbreviations: MD, mean difference; SD, standard deviation

Gene and SNP	Source	Iron parameter														Outcome					Risk of bias	Notes
		Ferritin	Transferrin	TS %	Hematocrit	Hemoglobin	MCV, MCH	Hepcidin	UIBC	TIBC	Serum Iron	Genotype frequency	Iron deficiency/ IDA risk	RBC count	Ancestry	Absolute Values and Mean Difference	Factor	Effect size	Other	P-value	JBI assessment	
**TF** rs1799852	Blanco-Rojo et al., 2011		x												Caucasian			Effect per A allele: -20.25	78% probability of association<br><br>variance explained: 5.5 %		7/8	
**TF** rs1799852	Baeza-Richer et al., 2015		x												Caucasian			*Linear regression for SNP (CT)*<br>Coef (95%CI): -25.45 (-39.29, -11.61)		0.0004	3/8	
**TF** rs1799852	Baeza-Richer et al., 2015				x										Caucasian	*Mean ± SD [%]*<br><br>CC (Wt): 38.371 ± 3.601<br>CT: 37.169 ± 3.804<br>MD(SD): -1.20 ± 5.24	Wt: 1<br>CT: 0.969	*Cohen's d* = - 0.23<br><br>*Linear regression for SNP (CT)*<br>Coef (CI 95%): -1.09 (-1.96, -0.23)		Mean values P = 0.018<br><br>Linear regression P = 0.014	3/8	
						x										*Mean ± SD [g/dL]*<br><br>CC (Wt): 12.826 ± 1.330<br>CT: 12.385 ± 1.496<br>MD ± SD: - 0.441 ± 2	Wt: 1<br>CT: 0.966	*Cohen's d* = - 0.22<br><br>*Linear regression for SNP (CT)*<br>Coef (CI 95%): -0.39 (-0.71, -0.07)		Mean values 0.022<br><br>Linear regression 0.019		

Gene and SNP	Source	Iron parameter													Ancestry	Outcome					Risk of bias	Notes
		Ferritin	Transferrin	TS %	Hematocrit	Hemoglobin	MCV, MCH	Hepcidin	UIBC	TIBC	Serum Iron	Genotype frequency	Iron deficiency/ IDA risk	RBC count		Absolute Values and Mean Difference	Factor	Effect size	Other	P-value	JBI assessment	
**BMP2**<br>rs235756	Al-Amer et al., 2021	x													Arab				*Genotype distribution, [n]*<br>Normal value<br>TT (Wt): 4<br>TC: 45<br>CC: 1<br>Low value<br>TT: 2<br>TC: 48<br>CC: 8<br>$X^2 = 5.65$	0.05	5/10	IDA:<br>Ferritin < 15ng/mL and Hb < 12g/dL<br><br>Iron deficiency:<br>Ferritin < 15ng/mL and Hb > 12g/dL
													x					*Odds and Risk for iron deficiency for CC*<br>OR (95% CI): 29.3 (1.494, 575.401)<br>RR (95%CI): 7.65 (0.549, 106.47)		0.026		

Footnotes
Effect directions refer to the minor allele T (A on reverse DNA strand); reference genotype: CC (wt)
Allele frequencies: C=0.891421, T=0.108579
Calculations to obtain Cohen's d are presented in the *Calculations* sheet

Gene and SNP	Source	Iron parameter														Outcome					Risk of bias	Notes
		Ferritin	*Transferrin*	*TS %*	*Hematocrit*	*Hemoglobin*	*MCV, MCH*	*Hepcidin*	*UIBC*	*TIBC*	*Serum Iron*	*Genotype frequency*	*Iron deficiency/ IDA risk*	*RBC count*	*Ancestry*	*Absolute Values and Mean Difference*	*Factor*	*Effect size*	*Other*	*P-value*	*JBI assessment*	
**BMP2** rs235756	Al-Amer et al., 2021											**x**			Arab				*Genotype distribution, [n]*<br>Control<br>TT (Wt): 4 (8%)<br>TC: 45 (90%)<br>CC: 1 (2%)<br>IDA<br>TT: 2 (3.45%)<br>TC: 48 (82.76%)<br>CC: 8 (13.79%)<br>$X^2 = 5.65$	0.05	5/10	
	Ji et al., 2018	**x**													Caucasian				"rs235756 was significantly associated with ferritin levels in male donors before and after adjusting for donor parameters"	adjusted P = 0.038 unadjusted P = 0.029	5/8	

Footnotes

Effect directions refer to minor allele C (G on reverse DNA strand); reference group: TT (wt) (AA on reverse DNA strand)

Allele frequencies: A=0.644149, C=0.000000, G=0.355851

Abbreviations: X2 Chi-square test

Gene and SNP	*Source*	***Iron parameter***													*Ancestry*	***Outcome***					***Risk of bias***	***Notes***
		Ferritin	*Transferrin*	*TS %*	*Hematocrit*	*Hemoglobin*	*MCV, MCH*	*Hepcidin*	*UIBC*	*TIBC*	*Serum Iron*	*Genotype frequency*	*Iron deficiency/ IDA risk*	*RBC count*		*Absolute Values and Mean Difference*	*Factor*	*Effect size*	*Other*	*P-value*	*JBI assessment*	
**Chromosome 2**<br>rs2698530	McLaren et al., 2011									x					Caucasian			GWAS<br>Coef: 20.72<br>Follow-up<br>Coef: 16.27	Meta-analysis<br>Variance explained: 3.0 %	GWAS: 5.01E-7 *<br>Follow-up: 0.032<br>Meta-analysis: 1.67E-7 **	9/10	* genome-wide corrected P = 0.17<br>** genome-wide corrected P = 0.055
				x														GWAS<br>Coef: -0.20<br>Follow-up<br>Coef: -0.02	Meta-analysis<br>Variance explained: 2.3 %	GWAS: 3.70E-7 *<br>Follow-up: 0.71<br>Meta-analysis: 3.51E-6		* genome-wide corrected P = 0.12
									x									GWAS<br>Coef: 28.75<br>Follow-up<br>Coef: 14.25	Meta-analysis<br>Variance explained: 3.0 %	GWAS: 5.96E-8 *<br>Follow-up: 0.11<br>Meta-analysis: 1.40E-7 *		* genome-wide corrected P = 0.02<br>** genome-wide corrected P = 0.046

Footnotes
Effect estimates refer to minor allele C; reference genotype: AA (wt)
Allele frequencies: A=0.757795, C=0.242205
Corr.: genome-wide corrected P-value
Genome-wide statistical significance was defined as P < 1.51E-7 (corrected P=0.05)

Source allele frequencies: *dbSNP, https://www.ncbi.nlm.nih.gov/snp/*
